# Anesthetic Strategy for 30 Radiotherapy Sessions in a Child With Autism Spectrum Disorder (ASD) and Severe Behavioral Dysregulation: A Case Report

**DOI:** 10.7759/cureus.106480

**Published:** 2026-04-05

**Authors:** Luiz Imbelloni, Anna Rivoli, Sylvio V Lemos Neto, Matheus V Santos, Antonio A SantaRosa, Norma P Modolo

**Affiliations:** 1 Anesthesiology, Instituto Nacional de Câncer (INCA), Retired, Rio de Janeiro, BRA; 2 Anesthesiology, Instituto Nacional de Câncer (INCA), Rio de Janeiro, BRA; 3 Genetics, Instituto Nacional de Câncer (INCA), Rio de Janeiro, BRA; 4 Anesthesiology, Botucatu Medical School, São Paulo State University (UNESP), Botucatu, BRA

**Keywords:** anesthesia, autism spectrum disorder, cancer, child, dexmedetomidine, ketamine

## Abstract

Autism spectrum disorder (ASD) refers to a spectrum of neurodevelopmental conditions characterized by challenges in social interaction and communication, which coexist with repetitive behaviors and narrowly focused interests. Autistic patients, particularly children, often present with heightened sensitivity, which must be considered when anesthesia is necessary. A six-year-old child with ASD was diagnosed with rhabdomyosarcoma of the left lower limb, and after surgical treatment, was indicated for 30 radiotherapy sessions. The routine technique for radiotherapy in children involves inhalation anesthesia with sevoflurane. After the first session, there were undesirable side effects, and it was decided to use sedation with clonidine as pre-anesthesia and ketamine and dexmedetomidine intranasally in the radiotherapy room. The remaining 29 sessions were all performed with this technique, achieving ease during radiotherapy and awakening without aggression in the post-anesthesia care unit (PACU). Clonidine is used as a pre-anesthetic medication in pediatrics due to its sedative and analgesic properties, providing preoperative sedation and preventing postoperative pain and vomiting. Ketodex is a combination of ketamine and dexmedetomidine. The combined use of ketamine and dexmedetomidine offers several clinical advantages, including cardiovascular stability, preservation of spontaneous ventilation, effective postoperative analgesia, and smooth recovery.

## Introduction

In Brazil, projections indicate approximately 7,930 new cancer diagnoses per year among children and adolescents between 2023 and 2025, yielding an incidence rate of about 134.8 cases per million [1]. Autism spectrum disorder (ASD) is a heterogeneous neurodevelopmental condition characterized by impairments in social communication and interaction, together with repetitive behaviors and circumscribed interests [2].

There is a potential link between ASD and different types of cancer. Some studies have found an overall increased risk of any type of cancer among individuals with ASD when compared with patients without ASD [3,4]. However, the potential risk may vary depending on specific conditions or syndromes [5].

Aggressive behavior occurs more frequently in individuals with ASD compared with those who have other developmental conditions [6]. These behaviors are linked to significant negative consequences for both children and their caregivers, such as reduced quality of life, heightened caregiver burden, and barriers to participation in educational and social support programs. Consequently, a range of pharmacological interventions has been employed to manage aggression in this population.

Several pharmacological agents are routinely employed to achieve sedation and analgesia during medical procedures. Ketamine, a long-established non-barbiturate and non-opioid anesthetic, provides dissociative anesthesia and analgesia, while dexmedetomidine exerts sedative, anxiolytic, amnestic, and analgesic effects, with the advantage of preserving respiratory drive and airway patency [7]. The combined administration of these agents, commonly referred to as Ketodex, has been applied in a variety of clinical settings. Clonidine is frequently used as a pediatric premedication because of its sedative and analgesic properties, as well as its ability to reduce perioperative pain and postoperative nausea and vomiting [8]. The intranasal route has been shown to provide more effective sedation in children when compared with oral chloral hydrate or oral midazolam [9]. Owing to its rapid onset, intranasal ketamine has also been utilized for anesthetic induction in pediatric patients and in individuals with significant cognitive or behavioral impairment, irrespective of age [10].

Radiation therapy is a cornerstone of oncologic treatment, using high-energy radiation to target malignant cells and achieve tumor reduction. A six-year-old child with ASD was scheduled for 30 radiotherapy sessions. During the first radiotherapy session, inhalational anesthesia with sevoflurane caused several recurrent effects, especially nausea and vomiting in the post-procedure period. To overcome these difficulties and avoid daily physical restraint, an alternative strategy was adopted. This involved the use of clonidine as a pre-anesthetic and the association of ketamine and dexmedetomidine via the intranasal route for serial radiotherapy in the treatment of lower limb embryonal rhabdomyosarcoma, forming the basis of this case report.

## Case presentation

The study was registered on Plataforma Brasil (Certificate of Presentation for Ethical Appreciation (CAAE): 93725925.8.0000.5274) and approved by the Instituto Nacional de Câncer (INCA) Research Ethics Committee (Approval Number: 8,117,457). Written informed consent was obtained from the patient’s parents after a detailed explanation of the procedure.

**Figure 1:**
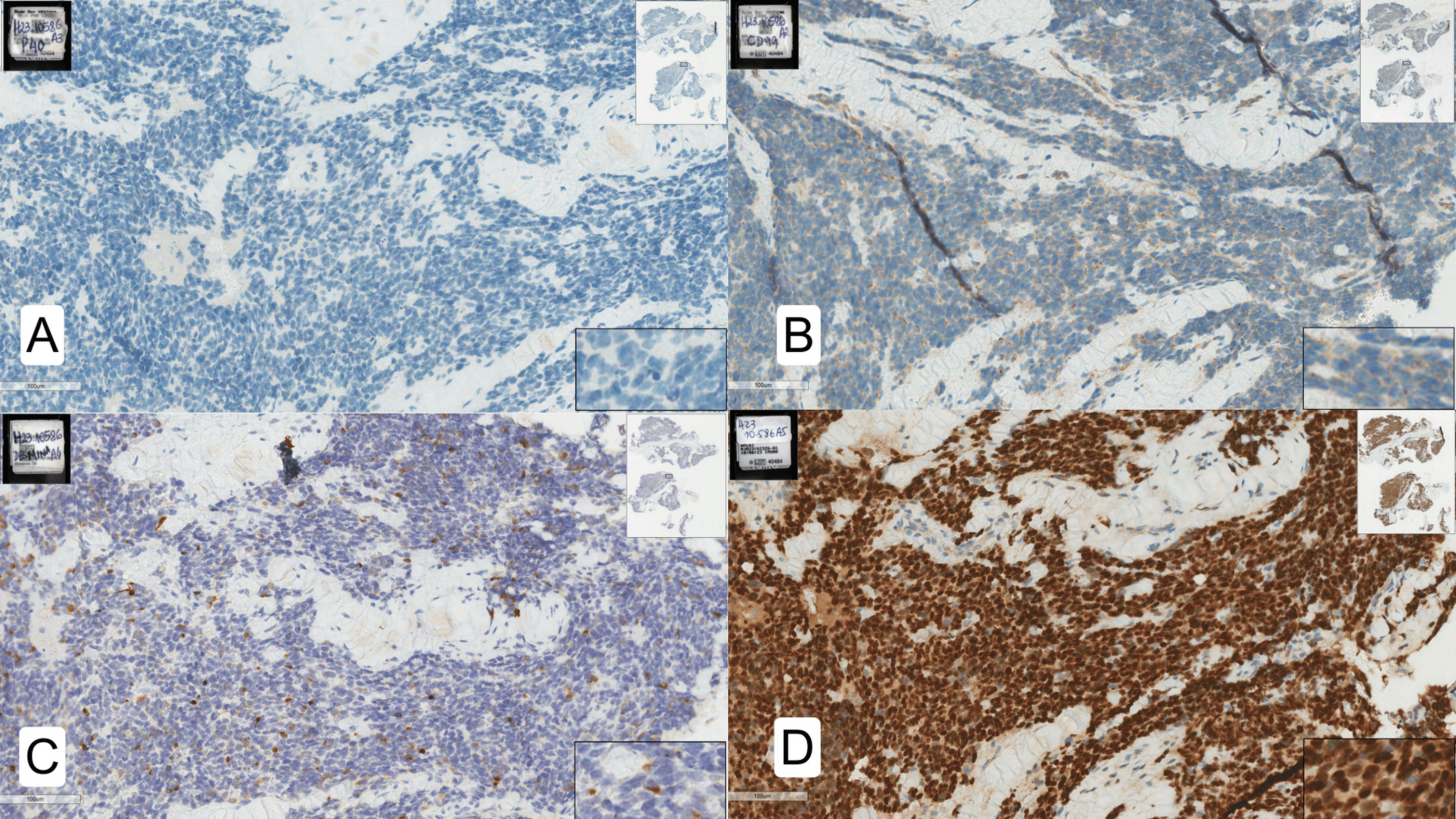
Histopathological and immunohistochemical findings. (A) Negative p40 immunostaining. (B) Negative CD99 immunostaining. (C) Positive desmin immunostaining. (D) Positive myogenin immunostaining.

**Figure 2:**
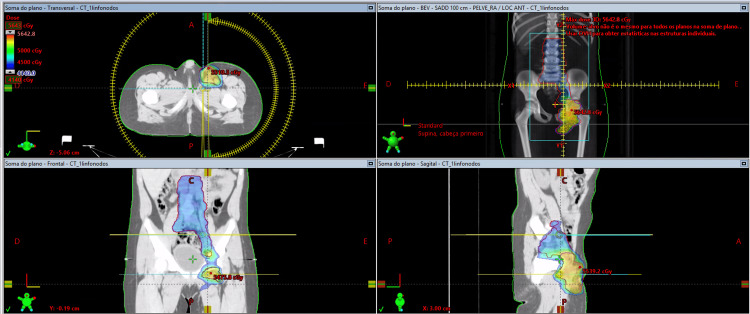
Radiotherapy planning. Treatment planning was performed using volumetric modulated arc therapy (VMAT) with 6 MV photon beams. The images demonstrate a conformal dose distribution to the primary tumor bed and regional lymph nodes, with adequate target coverage and surrounding tissue sparing.

The patient was a six-year-old male child, weighing 31 kg, diagnosed with embryonal rhabdomyosarcoma of the left lower limb. Histopathological examination revealed a malignant small round blue cell tumor consistent with embryonal rhabdomyosarcoma (Figure 1). Treatment included chemotherapy and surgery, followed by serial radiotherapy (Figure 2). The surgery consisted of the resection of the tumor in the left lower limb, along with lymphadenectomy in the left inguinal region.

Upon arrival in the radiotherapy suite, the patient was positioned on the treatment table, where standard monitoring, including pulse oximetry, was initiated. A previously placed 6.6-Fr catheter was present in the right internal jugular vein. No new venipuncture was performed on the upper limbs. The anesthesia technique for radiotherapy in children at the Department of Anesthesiology has always been inhalation anesthesia with sevoflurane, without venoclysis for general anesthesia, along with monitoring using a pulse oximeter [11].

The first of the 30 planned sessions was performed under general inhalational anesthesia with sevoflurane, with the need for physical restraint by the mother, resulting in recurrent adverse effects, notably post-anesthetic nausea and vomiting, as well as episodes of agitation and aggressive behavior observed in the post-anesthesia care unit (PACU). 

To increase safety and reduce physical and psychological stress, an alternative sedation protocol was implemented for the remaining 29 sessions. The protocol consisted of premedication with 150 µg of oral clonidine, administered approximately 60 minutes before each procedure, followed in the operating room (OR) by combined intranasal administration of dexmedetomidine 60 µg (2 µg/kg) and ketamine 30 mg (1 mg/kg), in doses compatible with institutional protocols for pediatric intranasal sedation. 

All procedures performed under this protocol were conducted without oxygen supplementation, with spontaneous ventilation maintained throughout the sessions. The mean duration of each session was 20 minutes. The depth of sedation was clinically monitored. No hemodynamic or respiratory complications were observed in any of these sessions. All radiotherapy sessions under this protocol were completed successfully, without the need for physical restraint, with minimal resistance from the patient, and without the need to supplement the technique or administer additional doses of the three drugs used.

## Discussion

Radiation therapy is a cornerstone in the treatment of cancer in children and is typically performed five days a week. The technique used at INCA was recently demonstrated in a pilot study with 25 patients undergoing inhalation anesthesia without venoclysis [11]. After attempting the usual inhalation technique in children with ASD, it was not successful. Therefore, a new technique using clonidine as a pre-anesthetic and the administration of ketamine and dexmedetomidine intranasally was developed, making it possible to perform 29 radiotherapy procedures successfully without the need for venipuncture and supplemental oxygen.

Clonidine is an α_2_-adrenergic agonist widely used in pediatric practice for multiple clinical purposes. Its applications include reducing postoperative emergence agitation, providing analgesic and anxiolytic effects, facilitating sedation, and assisting in the control of perioperative shivering [8]. Furthermore, clonidine has been associated with a decrease in the time required for anesthesia induction and a reduction in oxygen consumption. It also offers renal protection, helps to spare anesthetics, and provides myocardial protection [12].

In this six-year-old child, while undergoing the remaining 29 radiotherapy sessions, no oxygen supplementation was required, no agitation was observed, and there was a progressive improvement in the acceptance of the hospital environment, corroborating the safety of the anesthetic technique used. In oral and maxillofacial surgery, a systematic review showed that clonidine is an effective pre-anesthetic medication in reducing intraoperative blood loss [13]. Since the child had ASD and aggressiveness, the use of clonidine 60 µg before referral to the surgical center provided sufficient sedation for the intranasal instillation of ketamine with dexmedetomidine. As a dissociative anesthetic, ketamine remains chemically stable during storage at ambient temperature. The drug can induce increases in cerebral perfusion, intracranial pressure, and metabolic activity within the brain and can be used through several administration routes [14]. By selectively activating central α_2_-adrenergic receptors, dexmedetomidine decreases presynaptic catecholamine release. The combination of sedative, anxiolytic, and analgesic actions supports its role as an adjunctive agent for procedural sedation and sedation in critical care [7]. Because radiotherapy is not a painful procedure, its indication was excellent for this child with ASD, showing much better results than the use of inhalation anesthesia with sevoflurane.

A structured search of online bibliographic and medical literature databases was undertaken to retrieve studies evaluating the combined use of dexmedetomidine and ketamine for procedural sedation. Evidence suggests growing interest in this pharmacological association for both invasive and non-invasive procedures [15]. The dexmedetomidine-ketamine regimen may offer advantages over other commonly employed sedative strategies, including propofol, although direct comparative studies remain necessary to confirm its superiority [16]. In this child with ASD and aggressiveness, the inhalation technique could not be applied, and instead, oral clonidine and intranasal ketamine-dexmedetomidine were administered calmly and safely.

The ketamine-dexmedetomidine combination provided effective and reliable sedation across 29 radiotherapy sessions, with no observed adverse effects in the present case. Low doses of ketamine combined with low doses of dexmedetomidine can achieve effective analgesia and sedation while minimizing adverse effects. An editorial review reported that, in most studies, dexmedetomidine attenuated ketamine-related increases in heart rate, blood pressure, salivary secretion, and emergence reactions, whereas ketamine counterbalanced dexmedetomidine-induced bradycardia and hypotension [17]. This complementary pharmacodynamic interaction supports the characterization of the dexmedetomidine-ketamine combination as a safe and well-tolerated regimen [17]. In this context, the use of clonidine as premedication, followed by intranasal ketamine and dexmedetomidine, proves to be a safe and effective approach for delivering radiotherapy in children with ASD.

Aggression encompasses behaviors that involve a risk of harm to others and may occur through verbal expressions, such as threatening speech, or physical actions, such as striking, biting, or throwing objects. Research focusing on aggression in individuals with ASD remains insufficient [18]. One investigation enrolled 414 children with ASD and 243 clinic-referred children without ASD, with ages ranging from one to 21 years (with an average age of about seven years). Importantly, recruitment was not restricted by the presence of aggressive behavior [18]. Compared with the reference group, children with ASD were described as exhibiting lower levels of aggressive behavior and were more frequently characterized as reactive rather than proactive in nature. No association was identified between sex and the occurrence of aggression. In our case, the child was six years old and male. 

A recent review examining the relationship between ASD and selected cancer types explored potential pathways relevant to prevention and treatment [19]. The authors reported a possible association between ASD and certain malignancies, not through shared pathophysiological mechanisms, but rather via the involvement of specific genes implicated in cancers [19]. In another publication of 10 case reports on autism in the same journal, none of these cases of ASD were found to be associated with cancer [20]. Further, using the phrase "autism spectrum disorder case report and radiotherapy" on search sites such as PubMed, Medical Literature Analysis and Retrieval System Online (MEDLINE), Scientific Electronic Library Online (SciELO), Science, and Latin American and Caribbean Health Sciences Literature (LILACS), no articles were found regarding autism and cancer patients undergoing radiotherapy.

Case reports of anesthesia in ASD allow for an in-depth analysis of each individual case, demonstrating the complexity of this disorder and outlining the course and possible anesthetic techniques for radiotherapy. Although case reports do not provide the most solid evidence-based indications, they can assist in conducting exploratory analyses or generating intriguing hypotheses for investigating anesthetic techniques in these patients with a history of cancer.

## Conclusions

Although the causes of ASD are not fully understood, it is recognized as a multifactorial condition involving genetic and environmental influences, with significant prevalence in the pediatric population. Observations of shared biological pathways and occasional associations with other conditions have been described; however, such findings remain limited and should be interpreted with caution. Based on current knowledge of ASD, specific strategies may be employed during surgical procedures. Risk factors linking autism and cancer include genetic predispositions, environmental exposures, and underlying health conditions.

The child in this case, in addition to having embryonal rhabdomyosarcoma, was extremely aggressive, and the use of inhalational anesthesia with sevoflurane caused several deleterious effects in the postoperative period. Administration of clonidine as pre-anesthesia to reduce anxiety, combined with intranasal dexmedetomidine - known for its sedation profile without respiratory depression - and ketamine for pain control during positioning, provided an effective balance between comfort and safety during 29 out of 30 radiotherapy procedures. This case reinforces the importance of individualized sedation protocols for neurodivergent patients and opens the door for future studies that systematically evaluate this approach.
